# Undetectable ultrasensitive PSA after radical prostatectomy for prostate cancer predicts relapse-free survival

**DOI:** 10.1054/bjoc.2000.1474

**Published:** 2000-11-22

**Authors:** A P Doherty, M Bower, G L Smith, R Miano, E M Mannion, H Mitchell, T J Christmas

**Affiliations:** Department of Urology, Charing Cross Hospital, Fulham Palace Road, London; Department of Medical Oncology, Chelsea and Westminster Hospital, Fulham Road, London; Department of Histopathology, Charing Cross Hospital, Fulham Palace Road, London; Department of Medical Oncology, Charing Cross Hospital, Fulham Palace Road, London

**Keywords:** prostate cancer, PSA nadir, radical retropubic prostatectomy

## Abstract

Radical retropubic prostatectomy is considered by many centres to be the treatment of choice for men aged less than 70 years with localized prostate cancer. A rise in serum prostate-specific antigen after radical prostatectomy occurs in 10–40% of cases. This study evaluates the usefulness of novel ultrasensitive PSA assays in the early detection of biochemical relapse. 200 patients of mean age 61.2 years underwent radical retropubic prostatectomy. Levels ≤ 0.01 ng ml–1 were considered undetectable. Mean pre-operative prostate-specific antigen was 13.3 ng ml–1. Biochemical relapse was defined as 3 consecutive rises. The 2-year biochemical disease-free survival for the 134 patients with evaluable prostate-specific antigen nadir data was 61.1% (95% CI: 51.6–70.6%). Only 2 patients with an undetectable prostate-specific antigen after radical retropubic prostatectomy biochemically relapsed (3%), compared to 47 relapses out of 61 patients (75%) who did not reach this level. Cox multivariate analysis confirms prostate-specific antigen nadir ≤ 0.01 ng ml–1 to be a superb independent variable predicting a favourable biochemical disease-free survival (P < 0.0001). Early diagnosis of biochemical relapse is feasible with sensitive prostate-specific antigen assays. These assays more accurately measure the prostate-specific antigen nadir, which is an excellent predictor of biochemical disease-free survival. Thus, sensitive prostate-specific antigen assays offer accurate prognostic information and expedite decision-making regarding the use of salvage prostate-bed radiotherapy or hormone therapy. © 2000 Cancer Research Campaign http://www.bjcancer.com


*British Journal of Cancer* (2000) **83**(11), 1432–1436
© 2000 Cancer Research Campaign
doi: 10.1054/ bjoc.2000.1474, available online at http://www.idealibrary.com on IDEAL®

http://www.bjcancer.com

### Undetectable ultrasensitive PSA after radical prostatectomy for prostate cancer predicts relapse-free survival

**AP Doherty[1], M Bower[2], GL Smith[1], R Miano[1], EM Mannion[3], H Mitchell[4] and TJ Christmas[1]**

[1]Department of Urology, Charing Cross Hospital, Fulham Palace Road, London; [2]Department of Medical Oncology, Chelsea and Westminster Hospital, Fulham Road, London; [3]Department of Histopathology, Charing Cross Hospital, Fulham Palace Road, London; [4]Department of Medical Oncology, Charing Cross Hospital, Fulham Palace Road, London

Summary Radical retropubic prostatectomy is considered by many centres to be the treatment of choice for men aged less than 70 years with localized prostate cancer. A rise in serum prostate-specific antigen after radical prostatectomy occurs in 10–40% of cases. This study evaluates the usefulness of novel ultrasensitive PSA assays in the early detection of biochemical relapse. 200 patients of mean age 61.2 years underwent radical retropubic prostatectomy. Levels ≤ 0.01 ng ml–1 were considered undetectable. Mean pre-operative prostate-specific antigen was 13.3 ng ml–1. Biochemical relapse was defined as 3 consecutive rises. The 2-year biochemical disease-free survival for the 134 patients with evaluable prostate-specific antigen nadir data was 61.1% (95% CI: 51.6–70.6%). Only 2 patients with an undetectable prostate-specific antigen after radical retropubic prostatectomy biochemically relapsed (3%), compared to 47 relapses out of 61 patients (75%) who did not reach this level. Cox multivariate analysis confirms prostate-specific antigen nadir ≤ 0.01 ng ml–1 to be a superb independent variable predicting a favourable biochemical disease-free survival (P < 0.0001). Early diagnosis of biochemical relapse is feasible with sensitive prostate-specific antigen assays. These assays more accurately measure the prostate-specific antigen nadir, which is an excellent predictor of biochemical disease-free survival. Thus, sensitive prostate-specific antigen assays offer accurate prognostic information and expedite decision-making regarding the use of salvage prostate-bed radiotherapy or hormone therapy. © 2000 Cancer Research Campaign http://www.bjcancer.com

**Keywords:** prostate cancer; PSA nadir; radical retropubic prostatectomy

Radical retropubic prostatectomy (RRP) is a well-established treatment option for localized prostate cancer in patients less than 70 years of age. Unfortunately, in a proportion of these patients postoperative prostate-specific antigen (PSA) does not reach undetectable levels and biochemical relapse occurs (Pound et al, 1997; Graefen et al, 1999). Patients with organ-confined disease are more likely to have prolonged biochemical disease-free survival (BDFS). Consequently, presence of extracapsular disease (pT3) or positive margins have been used not only to predict outcome, but also to decide on adjuvant therapy (Partin et al, 1993; D'Amico et al, 1998). However, this criterion is not reliable in predicting biochemical relapse, and large numbers of patients (up to 70%) with positive margins do not progress (Partin et al, 1998; D'Amico et al, 1998). Moreover, patients with negative margins are not guaranteed to be free from progression, approximately 10% progress in some series (Catalona and Smith, 1994; D'Amico et al, 1998).

In an attempt to improve outcome from RRP, some have attempted to select patients with a good prognosis. Probability nomograms combining PSA levels, clinical stage and Gleason score (so-called 'Partin's Tables') have been advocated to predict pathological stage, and by inference, risk of progression (Partin et al, 1997). Although use of these tables has had the effect of improving BDFS figures, they are not accurate enough to confidently predict clinical or biochemical outcome for an individual patient. Consequently, some patients with unfavourable risk factors may be denied potentially curative treatment. Pre-operative criteria other than Partin's tables and postoperative criteria other than surgical margins are needed to reliably predict biochemical or clinical outcome.

PSA nadir measured with a sensitive assay offers the possibility of confidently predicting the removal of all significant PSA producing tissue within a few weeks of the operation. In contrast, histological evidence of relapse is usually not attained with more than 60% accuracy until the PSA rises above 2 ng $ml^{-1}$ (Connolly et al, 1996) which may not occur for many years after RRP. Thus, the earliest indicator of prostate cancer relapse after radical prostatectomy is a rising PSA. Most contemporary published series use a PSA level > 0.2 ng $ml^{-1}$ in defining undetectable levels of PSA (Partin et al, 1999), but levels ranging from 0.1–4 ng $ml^{-1}$ have also been used (Frazier et al, 1993; Dillioglugil et al, 1995; Feneley et al, 1996; Scardino, 1998). The advent and availability of a sensitive PSA assay (Yu and Diamandis, 1993) raises questions regarding the appropriateness of these unvalidated levels. Sensitive PSA assays have been reported as being clinically useful (Yu et al, 1995) but their role in early detection and prediction of biochemical relapses needs further assessment. Moreover, the benefits of early detection of relapse are still unknown; early detection of prostate cancer relapse might offer therapeutic advantages. Indeed, a recent study suggests that salvage external beam

*Received 23 December 1999*
*Revised 25 July 2000*
*Accepted 9 August 2000*

*Correspondence to:* AP Doherty

**Table 1** Pre and post-operative clinicopathological characteristics of 200 men with clinically localized prostate cancer

| Pre-operative criteria | Number of patients (%) | | | | | | | | | | |
|---|---|---|---|---|---|---|---|---|---|---|---|
| | Pathological Stage | | | | Specimen Gleason | | | | Margins | | |
| | T2 | T3a | T3b | N1 | 2–4 | 5–6 | 7 | 8–10 | Positive | Negative | Total |
| Biopsy Gleason | | | | | | | | | | | |
| 2–4 | 39 | 10 | 8 | 0 | 20 | 29 | 6 | 2 | 33 | 24 | 57 (28%) |
| 5–6 | 68 | 23 | 21 | 2 | 9 | 65 | 28 | 10 | 68 | 44 | 112 (56%) |
| 7 | 11 | 4 | 9 | 2 | 0 | 5 | 12 | 7 | 20 | 4 | 24 (12%) |
| 8–10 | 0 | 3 | 4 | 0 | 0 | 1 | 3 | 3 | 6 | 1 | 7 (4%) |
| PSA (ng/ml) | | | | | | | | | | | |
| <4 | 6 | 1 | 1 | 0 | 1 | 4 | 1 | 2 | 2 | 5 | 8 (4%) |
| 4–10 | 71 | 18 | 7 | 0 | 17 | 52 | 17 | 10 | 53 | 43 | 96 (48%) |
| 10.1–20 | 31 | 14 | 20 | 2 | 7 | 33 | 19 | 6 | 46 | 19 | 65 (32%) |
| >20 | 10 | 7 | 14 | 2 | 4 | 11 | 12 | 4 | 26 | 5 | 31 (16%) |
| Total | 118 (59%) | 40 (20%) | 42 (21%) | 4 (2%) | 29 (14%) | 100 (50%) | 49 (25%) | 22 (11%) | 127 (63%) | 73 (37%) | 200 |

radiotherapy (EBRT) to the prostate bed may be more effective if given at low PSA levels (Egawa et al, 1999).

A reasonable hypothesis is that postoperative PSA nadir levels measured by a sensitive PSA assay may provide useful prognostic information and expedite decision-making regarding the use of early salvage therapy. Thus, the objective of this study was to evaluate the role of sensitive PSA assays following radical prostatectomy.

### PATIENTS AND METHODS

A consecutive series of 200 patients of mean age 61.2 years (range 43–73) were selected for RRP and simultaneous pelvic lymph node dissection according to the following criteria. Patients had to have clinically organ-confined disease with histological confirmation of prostate cancer by transrectal ultrasound-guided needle biopsy or transurethral resection of prostate (TURP). Isotope bone scans were performed if the PSA was over 20 ng $ml^{-1}$ or the patient had musculoskeletal pain. All patients had a presumed life expectancy in excess of 10 years, and no significant comorbidity. Patients over the age of 70 years were only considered for RRP in exceptional circumstances. Patients were not refused surgery because of high biopsy Gleason scores or high pre-operative serum PSA, providing they fulfilled the other criteria, and had no evidence of extracapsular disease on CT scan or transrectal ultrasound. 9 patients had pre-operative radiotherapy and were excluded from subsequent analysis of biochemical disease-free survival (BDFS). 12 patients had pre-operative hormone therapy.

Serum PSA levels were measured with the Roche COBAS® CORE assay in the first 80 patients (lower detecting limit = 0.1 ng $ml^{-1}$). From 1997 onwards, 120 patients had measurements using the IMMULITE® ‘Third Generation’ sPSA assay (Diagnostic Products Corporation, DPC, Gwynedd, UK). This assay detects PSA down to below levels of 0.01 ng $ml^{-1}$. Interassay variation is negligible at ≥ 0.01 ng $ml^{-1}$. This low detection level is a consequence of the efficient centrifugal wash, which results in a low non-specific signal accompanied by a large specific signal afforded by the chemiluminescent label (Babson et al, 1991). Mean pre-op PSA for the whole group was 13.3 ng $ml^{-1}$ (range 0.18–59 ng $ml^{-1}$, median 10 ng $ml^{-1}$). (The patient with a pre-operative PSA of 0.18 ng/$mL^{-1}$ had been previously treated by EBRT.) Around half (48%) of patients had pre-op PSA levels of >10 ng $ml^{-1}$ (Table 1).

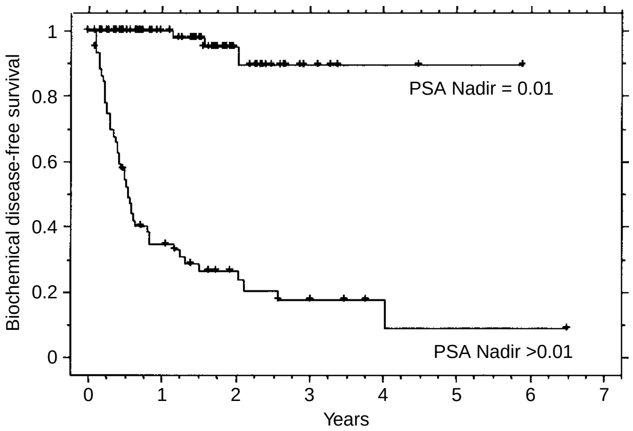

**Figure 1** Kaplan-Meier biochemical disease-free survival curves for patients with PSA nadir ≤0.01 ng $ml^{-1}$ and PSA nadir >0.01 ng $ml^{-1}$. Chi-square = 71.67, $P < 0.0001$

One surgeon between 1992–1999 performed all the operations via the retropubic approach. When possible, a nerve-sparing technique was used (Walsh et al, 1984). Two pathologists graded the specimens using the Gleason classification system (Gleason, 1977). The new TNM staging system was used for clinical and pathological staging. At initial biopsy, 31 (15.5%) patients had Gleason grade 7 or above. Clinicopathological data is shown in Table 1.

Patients were followed-up by PSA measurements every 3 months until a year and then every 6 months. Only 12 patients who are still alive have not had a sensitive PSA measurement.

Biochemical relapse was defined as 3 consecutive rising PSA values. PSA readings had to be at least 3 months apart. This is consistent with the definition proposed by ASTRO (Cox, 1997). An undetectable PSA nadir is defined as ≤0.01 ng $ml^{-1}$.

The log rank test was used to analyse differences in survival duration. Multivariate analysis of prognostic variables was performed by the Cox proportional hazards model. In addition, survival duration from the date of surgery to death from any cause and survival free of biochemical relapse were estimated by the Kaplan-Meier method.

*© 2000 Cancer Research Campaign* 

**Table 2** Predictors of BDFS. Univariate analysis

| Variable | Chi Squared | *P* value |
|---|---|---|
| Age | 0.001 | 0.97 |
| Biopsy Gleason score | 2.7 | 0.099 |
| Seminal vesicle invasion | 10.3 | 0.0013 |
| Positive surgical margins | 12.5 | 0.0004 |
| Gleason of surgical specimen | 14.1 | 0.0002 |
| Extracapsular spread | 16.9 | <0.0001 |
| Pre-operative PSA | 18.4 | <0.0001 |
| PSA nadir ≤0.01 ng ml$^{-1}$ | 71.6 | <0.0001 |

**Table 3** Predictors of BDFS. Multivariate analysis.

| Variable | Chi square | *P* value |
|---|---|---|
| Gleason specimen sum | 3.551 | 0.0595 |
| Pre-operative PSA | 4.308 | 0.0379 |
| Surgical margin positive | 4.703 | 0.0301 |
| PSA nadir ≤ 0.01 ng mL$^{-1}$ | 26.910 | <0.0001 |

**Table 4** Probability tables enumerating the likelihood of reaching undetectable PSA (≤0.01 ng/ml) based on pre-operative criteria; biopsy Gleason and PSA. Note that, in view of small numbers, Gleason 7 scores have been included with poorly differentiated tumours graded 8–10.

| | Charing Cross tables: numbers who achieved PSA nadir ≤ 0.01 ng/ml | | | |
|---|---|---|---|---|
| | Biopsy Gleason | | | |
| **PSA (ng/ml)** | **2–4** | **5–6** | **7–10** | **Totals** |
| ≤ 10 | 10/17 (59%) | 33/46 (72%) | 2/3 (66%) | 45/66 (68%) |
| >10–20 | 11/18 (55%) | 8/21 (38%) | 3/9 (33%) | 22/48 (46%) |
| >20 | 5/9 (55%) | 1/9 (11%) | 0/2 (0%) | 6/20 (30%) |
| Totals | 26/44 (59%) | 42/76 (55%) | 5/14 (36%) | 73/134 (55%) |

### RESULTS

Clinicopathological details are outlined in Table 1. 4 patients had histopathologically proven nodal involvement, despite CT scans showing normal pelvic lymph node morphology. 82 (41%) had pT3 disease despite being considered clinically organ-confined. 49 (25%) had pathological specimen Gleason scores ≥7.

Median follow-up was 1.7 years (maximum 6.5 years). Overall 2-year survival was 98.3% (95% CI: 96.4–100%). 4 patients have died, one of metastatic prostate cancer (5 years after RRP) and 3 from other causes. Overall prostate cancer specific survival was 100% at 2 years.

Patients who did not have evaluable nadirs. These included those who had had prior radiotherapy ($n$ = 9), early deaths ($n$ = 3) and adjuvant postoperative EBRT for positive margins ($n$ = 4). Another 44 patients had nadirs below the lower detection limit for the Roche COBAS® CORE system. 13 patients have not yet reached their nadir. PSA nadir data was therefore analysed in 134 patients. 73 patients achieved an undetectable PSA nadir (≤ 0.01 ng ml$^{-1}$). The median time to reach PSA nadir ≤ 0.01 ng ml$^{-1}$ was 10.4 weeks (range 2.6–214 weeks).

Of the 200 patients, those who had pre-operative ($n$ = 9) or adjuvant EBRT ($n$ = 4) or early deaths ($n$ = 3) were excluded from analysis of BDFS. Of the remaining 184 patients biochemical relapse occurred in 72 (39%). Two-year BDFS was 68.2% (61.5–75.9%).

Of the 134 patients with evaluable PSA nadir data recorded, 49 (36.5%) have failed biochemically. This gives a 2-year BDFS of 61.1% (95% CI: 51.6–70.6%). Out of the 73 patients who have reached an undetectable nadir, only 2 have failed (3%); this compares with 47 relapses out of the 61 patients (76%) who did not reach undetectable levels. Patients who achieve undetectable PSA nadir have significantly greater BDFS (Log Rank Test $P < 0.0001$). The 2-year BDFS for PSA nadir >0.01 ng ml$^{-1}$ was 26.4% (95% CI: 14.4–38.4%) compared to those with a PSA nadir ≤0.01 ng ml$^{-1}$ of 94.8% (95% CI: 87.5–100%). Figure 1 shows BDFS for nadir evaluable patients by comparing achievement of undetectable PSA nadir.

Univariate analysis of variables predictive of BDFS showed no significant association with age or biopsy Gleason score (Table 2). However, seminal vesicle invasion ($P$=0.0013), surgical margins ($P$=0.0004), specimen Gleason score ($P$=0.0002), extracapsular spread ($P \leq 0.0001$), pre-operative PSA ($P < 0.0001$) and undetectable PSA nadir ($P < 0.0001$) were prognostic variables. All these variables were entered into a Cox multivariate model to determine which variables were of independent prognostic significance. Table 3 shows that only undetectable PSA nadir ($P \leq 0.0001$), positive surgical margins ($P = 0.03$) and pre-operative PSA ($P = 0.04$) were independent variables predicting a favourable BDFS.

On the basis of the finding that PSA nadir is the most significant predictor of BDFS, a model to predict PSA nadir and hence BDFS was constructed using pre-operative variables to aid patient selection. The nomograms derived from the Charing Cross dataset is shown in Table 4. Patients with low-grade tumours had a better outcome. Moreover, the deleterious effect of increasing levels of pre-op PSA level was less marked in well-differentiated tumours.

### DISCUSSION

Radical retropubic prostatectomy is a popular treatment for men less than 70 years with clinically localized prostate cancer. After RRP, a proportion of patients will have pathological non-organ-confined disease, often with positive margins. This has led to a quest for accurate pre-operative markers with the aim of increasing the identification of patients with organ-confined

 © 2000 Cancer Research Campaign disease. Patients with organ-confined disease are more likely to have complete excision of the tumour, which should result in cure. Thus, in previous large-scale studies of outcome after radical prostatectomy, attention has largely focused upon histology of the RRP specimen to define success of surgery. The overall incidence of positive surgical margins after radical prostatectomy has been reported to range from 7–45% in patients with clinically localized cancer prior to surgery (Eggleston and Walsh, 1985; Catalona and Bigg, 1990). The accuracy with which surgical margins can predict outcome is reasonably good, especially if combined with grade. Pound et al found that in men with capsular penetration with Gleason score 2–6, the status of the surgical margin had a significant effect on outcome at 10 years. However, the outcome in both groups was good with the likelihood of an undetectable PSA at 10 years with a negative margin being 89% and 72% for men with a positive margin (Pound et al, 1997). Thus, biochemical relapse is not always a consequence of positive surgical margins (D'Amico et al, 1998). Opting for adjuvant therapy on the basis of a positive surgical margin status alone may result in unnecessary treatment of the patient.

This lack of reliability may be because of the heterogeneity of a positive margin (Rosen et al, 1992). Positive apical margins are apparently less clinically significant than positive posterior-lateral margins (Fesseha et al, 1997). The pathological determination of margin positive disease also requires its discrimination from anatomical artefact, remembering that the prostate is in direct contact with the rectum and pelvic sidewall with little or no surrounding connective tissue elsewhere. Despite these limitations, we identified positive margins as an independent predictor of BDFS in our series.

Postoperative PSA readings are regarded by many to be the best criteria for determining tumour-free status. However, levels less than 0.2 ng $ml^{-1}$ are generally considered of little clinical value, despite some large clinical studies demonstrating early detection of relapse with sensitive PSA assays (Yu et al, 1997). Indeed, the most common level below which PSA is considered 'undetectable' is 0.2 ng $mL^{-1}$. In a recent study of the natural history of PSA elevation following RRP, 23% of patients with 'undetectable' PSA (defined as less than 0.2 ng $ml^{-1}$) biochemically relapsed after 5 years (Pound et al, 1999). This suggests that PSA levels <0.2 ng $ml^{-1}$ are often associated with the presence of residual prostate cancer.

In this study, sensitive PSA was used to measure nadir post RRP. A level of greater than 0.01 ng $ml^{-1}$ was found to be more accurate than any other criteria for prediction of subsequent biochemical relapse. Measuring the PSA to low levels minimizes the risk of falsely reassuring patients of surgical cure. The most sensitive PSA assay ever reported was by Ferguson et al (1996). Such ultrasensitive assays which detect PSA to levels less than 0.01 ng $ml^{-1}$ may subsequently be shown to be even more accurate in predicting biochemical outcome.

It is not possible to exclude some of the difference on BDFS accounted for by prolonged lag time in patients who achieve undetectable nadirs. However, the contour of the BDFS Kaplan-Meier Curve (Figure 1) for patients with undetectable nadir suggests that a plateau is achieved after 2 years. It is anticipated that prolonged follow-up of this series of patients will confirm this.

In addition sensitive PSA assays can detect disease relapse by consecutive PSA rises from very low levels (Witherspoon, 1997). Concern regarding the use of sensitive assays in this way is the potential lack of specificity in diagnosing prostate cancer recurrence. This anxiety relates to the fact that extraprostatic sources of PSA production are known to exist (Kamoshida and Tsutsumi, 1990; Yu et al, 1995). Nevertheless, this is a very small contribution to serum PSA (Oesterling et al, 1996) and is believed not to complicate the interpretation of monitoring since these sources contribute a stable amount of PSA in the serum, which does not change with time. Similarly, although PSA values rise as part of the natural history in benign as well as malignant prostatic disease, this has only been reported in patients with the prostate in-situ. For example, patients with benign prostatic hyperplasia typically have a PSA rise of 0.2 ng $ml^{-1}$ $year^{-1}$ (Collins et al, 1993). It is not necessarily the case that residual benign prostatic tissue after RRP will have the similar PSA kinetics. Recent papers have suggested the use of PSA doubling time to predict malignant potential (Patel et al, 1997), but even this parameter is of questionable value. Our data suggest that all patients who relapse biochemically continue to have an unremitting rise, and this occurs whether biochemical relapse is detected early (with sensitive assays) or late.

Another reason for the limited enthusiasm for sensitive assays is the lack of proven benefit from the use of early salvage external beam radiotherapy (EBRT). Critics claim that salvage EBRT to the prostate bed risks over-treating patients with rising PSA due to either undiagnosed metastatic disease or benign disease. However, potential benefits do exist for salvage EBRT. In a recent study investigating the outcomes of patients who received salvage EBRT after radical retropubic prostatectomy, 40% of the 32 patients enrolled had 'undetectable' post-radiotherapy PSA values at an average follow-up of 12 months. Moreover, the best outcomes were associated with patients who had the lowest pretreatment PSA levels (Egawa et al, 1999).

Probability tables as shown in this paper provide information on the likelihood of achieving an undetectable PSA nadir based on pre-op PSA and biopsy Gleason score. Although this is useful, the ability to predict accurately biochemical outcome in an individual patient prior to surgery is still unattainable. Reliably selecting patients for RRP who will not relapse biochemically after surgery continues to be a problem. However, ultrasensitive PSA assays are of benefit in the postoperative detection of relapse which is now known to be advantageous in planning adjuvant therapy.

### CONCLUSIONS

The role of RRP has been controversial in the UK partly because of the scepticism regarding the effectiveness of the procedure. Undoubtedly, not all patients undergoing RRP are cured of prostate cancer and patient selection is an important factor. Patients are keen to know if they have been cured of cancer after RRP. Until recently, to predict this we have been reliant upon histology alone. However, ultrasensitive PSA assays now enable us more accurately to advise patients of their chance of PSA relapse within 2 months of surgery and appears to be a reliable predictor of cure of prostate cancer.

### REFERENCES

Babson AL, Olson DR, Palmieri T, Ross AF, Becker DM and Mulqueen PJ (1991) The Immulite assay tube: a new approach to heterogeneous ligand assay. *Clin Chem* **37**: 1521–1522

Catalona WJ and Bigg SW (1990) Nerve-sparing radical prostatectomy: evaluation of results after 250 patients. *J Urol* **143(3)**: 538–543

© 2000 Cancer Research Campaign

Catalona WJ and Smith DS (1994) 5-year tumour recurrence rates after anatomical radical retropubic prostatectomy for prostate cancer. *J Urol* **152**: 1837–1842
Catalona WJ, Smith DS, Ratliff DS and Basler JW (1993) Detection of organ-confined prostate cancer is increased through prostate-specific antigen-based screening. *JAMA* **270(8)**: 948–954
Collins GN, Lee RJ, McKelvie GB, Rogers AC and Hehir M (1993) Relationship between prostate-specific antigen, prostate volume and age in the benign prostate. *Br J Urol* **71**: 445–450
Connolly JA, Shinohara K and Presti JC (1996) Local recurrence after radical prostatectomy: characteristics in size, location, and relationship to prostate-specific antigen and surgical margins. *J Urol* **47**: 225–231
Cox JD (1997) American Society for Therapeutic Radiology and Oncology (ASTRO). Consensus Statement: Guidelines for PSA following radiation therapy. *Int J Radiat Oncol Biol Phys* **37**: 1035–1041
D'Amico AV, Whittington R, Malkowicz SB, Fondurulia J, Chen MH, Tomaszewski JE and Wein A (1998) The combination of pre-operative prostate-specific antigen and post-operative pathological findings to predict prostate-specific antigen outcome in clinically localised prostate cancer. *J Urol* **160**: 2096–2101
Dillioglugil O, Leibman BD and Kattan M (1995) Hazard rates for progression determined by PSA, after radical prostatectomy for T1–T2 prostate cancer. *J Urol* **153**: 391A
Egawa S, Matsumoto K, Suyama K, Soh S, Kuwao S and Iwamura M (1999) Limited suppression of prostate-specific antigen after salvage radiotherapy for its isolated elevation after radical prostatectomy. *Urology* **53(1)**: 148–154
Eggleston JC and Walsh PC (1985) Radical prostatectomy with preservation of sexual function: pathological findings in the first 100 cases. *J Urol* **134(6)**: 1146–1148
Feneley MR, Gillatt DA, Hehir M and Kirby RS (1996) A review of radical prostatectomy from three centres in the UK: clinical presentation and outcome. *Br J Urol* **78**: 911–920
Ferguson RA, Yu H, Kalyvas M, Zammit S and Diamandis EP (1996) Ultrasensitive detection of prostate-specific antigen by a time-resolved immunofluorometric assay and the Immulite immunochemiluminescent third-generation assay: potential applications in prostate and breast cancer. *Clin Chem* **42**: 675–684
Fesseha T, Sakr W, Grignon D, Banerjee M, Wood DP and Pontes JE (1997) Prognostic implications of a positive apical margin in radical prostatectomy specimens. *J Urol* **158**: 2176
Frazier HA, Robertson JE and Humphrey PA (1993) Is prostate-specific antigen of clinical importance in evaluating outcome after radical prostatectomy? *J Urol* **149**: 516–518
Gleason DF (1977) Histologic grading and clinical staging of carcinoma of the prostate. In: Tannenbaum M (ed) *Urologic Pathology*. pp 171–198. Pa: Lea & Febiger, Philadelphia.
Graefen M, Noldus J, Pichlmeier U, Haese A, Hammerer P, Fernandez S, Conrad S, Henke R, Huland E and Huland H (1999) Early prostate-specific antigen after radical retropublic prostatectomy: Prediction on the basis of preoperative and postoperative tumor characteristics. *Eur Urol* **36**: 21–30
Kamoshida S and Tsutsumi Y (1990) Extraprostatic localisation of prostatic acid phosphatase and prostate-specific antigen: distribution in cloacogenic glandular epithelium and sex-dependent expression in human anal gland. *Human Pathol* **21**: 1108–1111
Oesterling JE, Tekchandani AH, Martin SK, Bergstralh EJ, Reichstein E, Diamandis EP, Yemeto C, Stamey TA (1996) The periurethral glands do not significantly influence the serum prostate specific antigen concentration. *J Urol* **155(5)**: 1658–1660
Partin AW, Carter HB and Chan DW (1990) Prostate-specific antigen in the staging of localised prostate cancer: influence of tumour differentiation, tumour volume and benign hyperplasia. *J Urol* **143**: 747–752
Partin AW, Pound CR and Clemens JQ (1993) Serum PSA after anatomic radical prostatectomy. *Urol Clin North Am* **20(4)**: 713–724
Partin AW, Kattan MW, Subong EN, Walsh PC, Wojno KJ, Oesterling JE, Scardino PT and Pearson JD (1997) Combination of prostate-specific antigen, clinical stage, and Gleason score to predict pathological stage of localised prostate cancer. Multi-institutional update. *JAMA* **277(18)**: 1445–1451
Patel A, Dorey F, Franklin J and Dekernion (1997) Recurrence patterns after radical retropubic prostatectomy: Clinical usefulness of prostate-specific antigen doubling times and log slope prostate-specific antigen. *J Urol* **158**: 1441–1445
Pound CR, Partin AW, Epstein JI and Walsh PC (1997) PSA following anatomical radical prostatectomy: patterns of recurrence and cancer control. *Urol Clin N Am* **24(2)**: 395–406
Pound CR, Partin AW, Eisenberger MA, Chan DW, Pearson JD and Walsh PC (1999) Natural history of progression after PSA elevation following radical prostatectomy *JAMA* **281**: 17 1591–1597
Rosen MA, Goldstone L, Lapin S, Wheeler T and Scardino PT (1992) Frequency and location of extracapsular extension and positive surgical margins in radical prostatectomy specimens. *J Urology* **148**: 331–337
Scardino PT (1998) Clinical and pathological significance of the level and extent of capsular invasion in clinical stage T1–2 prostate cancer. *Hum Pathol* **29**: 856–862
Walsh PC and Mostwin JL (1984) Radical prostatectomy and cystoprostatectomy with preservation of potency. Results using a new nerve-sparing technique. *Br J Urol* **56(6)**: 694–697
Witherspoon LR (1997) Early detection of cancer relapse after prostatectomy using very sensitive prostate-specific antigen measurements. *Br J Urol* **79** Suppl 1: 82–86
Yu H and Diamandis EP (1993) Ultrasensitive time-resolved immunofluorometric assay of prostate-specific antigen in serum and preliminary clinical studies. *Clin Chem* **39**: 2108–2114
Yu H and Diamandis EP (1995) Measurement of serum prostate-specific antigen levels in women and in prostatectomised men with an ultrasensitive immunoassay technique. *J Urol* **153**: 1004–1008
Yu H and Diamandis EP, Prestigiacomo AF and Stamey TA (1995) Ultrasensitive assay of prostate-specific antigen used for early detection of prostate cancer relapse and estimation of tumor-doubling time after radical prostatectomy. *Clin Chem* **41(3)**: 430–434
Yu H, Diamandis EP, Wong P, Nam R and Trachtenberg J (1997) Detection of prostate cancer relapse with prostate specific antigen monitoring at levels of 0.001 to 0.1 mG./L. *J Urol* **157**: 913–918

 *© 2000 Cancer Research Campaign*
